# Intragenomic rDNA Variants Identified in *Rotylenchulus borealis* and *R. macrodoratus* Populations Associated with Olive Groves in Italy

**DOI:** 10.3390/plants15101423

**Published:** 2026-05-07

**Authors:** Alessio Vovlas, Alberto Troccoli, Elena Fanelli, Ebunoluwa Ijeoma Ajobiewe, Francesca De Luca

**Affiliations:** 1 Istituto per la Protezione Sostenibile delle Piante-CNR, Via Amendola 122/D Bari, 70126 Bari, Italy; alessiovovlas@cnr.it (A.V.); alberto.troccoli@cnr.it (A.T.); elena.fanelli@cnr.it (E.F.); ebunoluwaijeoma.ajobiewe@unibas.it (E.I.A.); 2 Department for Humanistic, Scientific and Social Innovation, University of Basilicata, Via Lanera, 20, 75100 Matera, Italy

**Keywords:** Bayesian inference, cytochrome c oxidase subunit, D2-D3 expansion domains, ITS, ribosomal variability, *Rotylenchulus*

## Abstract

Plant-parasitic reniform nematodes of the genus *Rotylenchulus* are semi-endoparasites of herbaceous and woody plants occurring in regions with Mediterranean, tropical, and subtropical climates. In the present study, the occurrence of reniform nematodes in the rhizosphere of three olive orchards in Central Italy and six in Sicily (Italy) was investigated. Two *Rotylenchulus* species were recovered in olive groves in Central Italy, and no *Rotylenchulus* species were found in Sicily. Using the integrative taxonomy approach, combining morphological, molecular and multivariate morphological analyses, the two species were identified as *R. borealis* and *R. macrodoratus*. The D2-D3 sequencing of four individual specimens of Italian *R. macrodoratus* revealed the occurrence of unique haplotypes differing in nucleotide composition each other. Interestingly, the sequencing of different ITS clones from an individual specimen of the Italian *R. borealis* showed two ITS paralogs differing in length and nucleotide sequence compared with those of other specimens from the same population and showing higher similarity with those from other populations. Phylogenetic analyses, based on D2-D3 expansion domains of the 28S rRNA gene, ITS, and mitochondrial COI, confirmed the high level of ribosomal variability in both species and the occurrence of new mitochondrial haplotypes for the COI. The present study confirms the occurrence of high variability in *Rotylenchulus* genus and the existence of variant gene copies in the same specimen that could contribute to the survival of these species in different environments.

## 1. Introduction

Olive cultivation is an important part of the agriculture in the Mediterranean Basin and in Italy [1,2]. The climate of Italy is very different from north to south, creating diverse microclimates for olive groves. Olive production is significantly impacted by climate change, with risks including droughts, extreme temperatures, soilborne pathogens and pests. The authors of [1] reported that traditional olive orchards contained low levels of plant parasitic nematodes in the rhizosphere, while modern olive production seems to affect plant parasitic nematode composition, increasing nematode genera such as *Meloidogyne*, followed by *Pratylenchus*, *Helicotylenchus*, *Rotylenchulus*, *Tylenchorhynchus*, *Heterodera* and *Tylenchulus* [3,4]. In Italy, to reduce production costs, super-high density olive groves, along with traditional and organic ones, have been introduced, and the study of Landi et al. [5] referred to the same occurrence of plant parasitic nematode composition as shown by Castillo et al. [2]. In Italy, *Meloidogyne* and *Pratylenchus* are frequently found in olive orchards; however, *Rotylenchulus* species have been also reported in several olive-growing areas, particularly in central and southern regions, where environmental conditions favor nematode development. The genus *Rotylenchulus* (reniform nematodes) is not the most common or damaging nematode associated with olive orchards, but it can be relevant under certain conditions (drought, poor soil fertility and high population density). It has a broad host range affecting more than 350 plant species and is distributed mainly in regions with temperate, tropical, and subtropical climates. The genus currently comprises ten confirmed species widely distributed and with different pathogenicities. However, their economic importance is poorly investigated, as tree declines are often attributed to drought, nutrition, or other soil issues. In Italy, *R. macrodoratus* Dasgupta, Raski & Sher, 1968, *R. borealis* (=*macrosoma*) Loof & Oostenbrink 1962, and *R. reniformis* Linford & Oliveira, 1940 have been reported [2,6,7,8,9,10].

*Rotylenchulus macrodoratus* is known to infect several plant hosts, including grapevine, almond, wild and cultivated olive orchards in Apulia, Calabria, and Sicily (South Italy) [6,11,12,13], while *R. borealis* was mainly isolated in corn and cultivated olive in North Italy and in wild and cultivated olive orchards in Sothern Italy [14]. *Rotylenchulus reniformis* was mainly found in horticultural crops and weeds and less common in olive orchards in Italy. As *Rotylenchulus* species show conserved gross morphology, molecular identification is important to separate species with different pathogenicities and to adopt sustainable control. Recently, the authors of [15] have proposed the synonymization of *R. macrosoma* with *R. borealis* based on the close similarity of main diagnostic morphological characters and the clustering of *R. borealis* and *R. macrosoma* populations in the phylogenetic trees based on D2-D3 domain of the 28S rRNA gene, ITS1 and COI markers.

The present study aims to identify and characterize two *Rotylenchulus* populations isolated in traditional and organic olive orchards in central Italy by using the D2-D3 expansion domains, as well as the ITS and COI markers. Sequencing of ribosomal and mitochondrial markers, phylogenetic relationships, and multivariate analyses among Italian *Rotylenchulus* spp. and those from databases were conducted to investigate the genetic intra- and interspecific variability. As no detailed morphological analyses had been carried out of Italian *R. borealis* and *R. macrodoratus*, morphometric studies are also presented herein.

## 2. Results

For each sampling point, four subsamples were collected from nearby olive trees, taken at a depth of 20–40 cm and at a distance of approximately 30–50 cm from the trunk, corresponding to the active root zone. The subsamples were pooled to obtain a single composite soil sample per tree.

Seven soil samples collected from the rhizosphere of cultivated olive groves in Lazio (four samples) and Tuscany regions (three samples) (Central Italy) contained two reniform nematode species in high densities (70 nematodes/100 cm^3^ soil and 85 nematodes/100 cm^3^ soil, respectively). Morphological and molecular characterization identified these species as *R. borealis* (from Lazio) and *R. macrodoratus* (from Tuscany). Both species have already been reported in Italy on corn and olive [9,11,13,16,17]. Morphometrics of the two species are almost identical to populations previously described from South and Central Italy in olive orchards, with minor differences. As no detailed morphometrics have been carried out of Italian *R. borealis* and *R. macrodoratus*, we will report them below.

### 2.1. Morphology and Morphometrics

#### 2.1.1. Rotylenchulus Borealis Loof & Oostenbrink 1962 [7]

**Figure 1:**
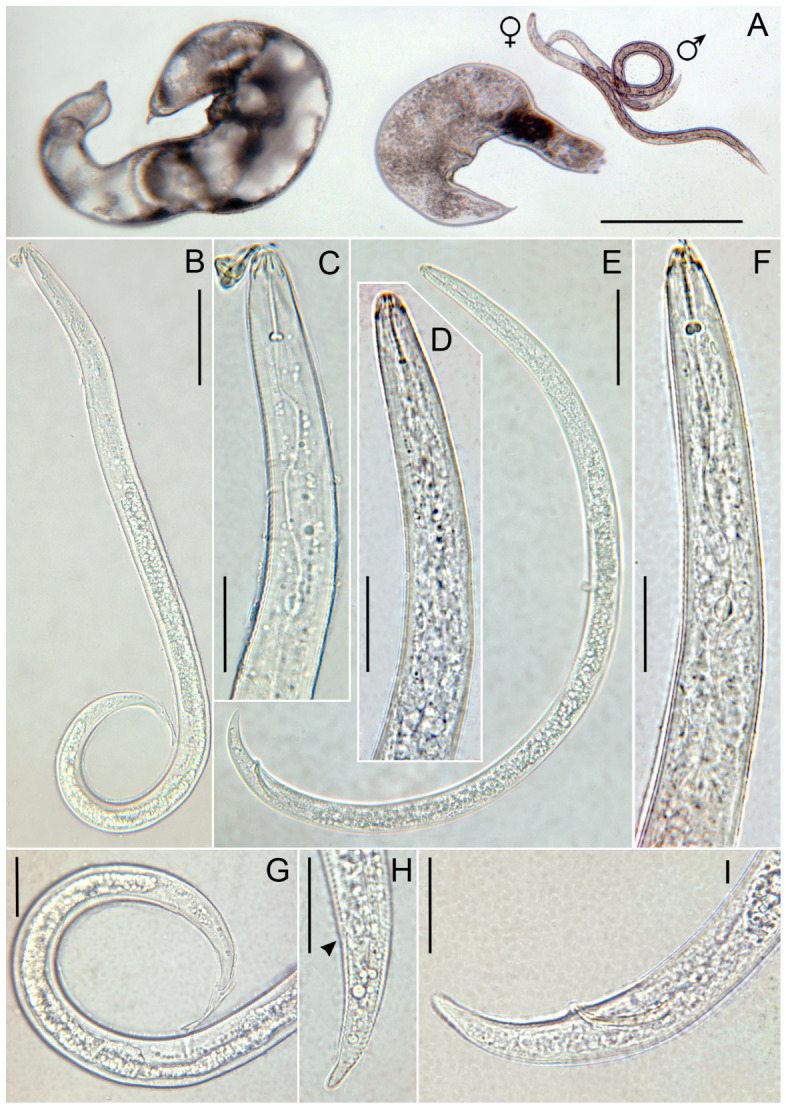
Light micrographs of *Rotylenchulus borealis*. (**A**): Entire body of two mature females (left), one immature female, and one male; (**B**): entire body of immature female; (**C**): part of pharyngeal region of B; (**D**): male pharyngeal region; (**E**): male entire body; (**F**): female pharyngeal region; (**G**): immature female vulval and tail region; (**H**): immature female tail (anus arrowed); (**I**): male tail. (Scale bars: (**A**) = 200 µm; (**B**,**E**) = 50 µm; (**C**,**D**,**F**–**I**) = 20 µm).

**Figure 2:**
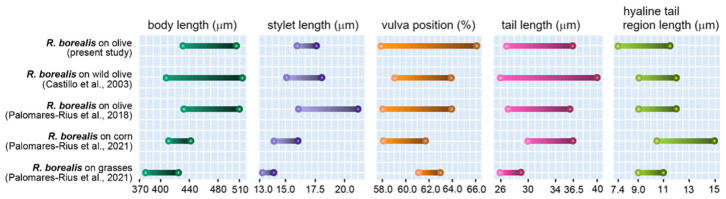
Schematic representation of the range of variations of five main diagnostic characters of immature females among *Rotylenchulus borealis* populations compared with populations previous described in the literature: *R. borealis* on wild olive [1], *R. borealis* on olive [9], *R. borealis* on corn and grasses [15].

The main diagnostic morphological traits of eleven immature females of the Italian *Rotylenchulus* population recovered on olive in Central Italy were compared (Figure 1, Table S1) with those of immature females of Spanish populations of *R. borealis* (=*R. macrosoma*) from wild olive, a Greek population from cultivated olive, an Italian population from corn and paratypes of the original Dutch population from grasses. Figure 2 reports the schematic representation of the range of variations of the five main diagnostic characters among immature females of *R. borealis* populations.

The recent synonymy proposed by [15] of *R. macrosoma* with *R. borealis*, which the authors of the present study agree with, has expanded the morphometric range of the species. Nevertheless, our population is largely like conspecific populations, which are compared in Table S1. Small differences concerned the total body length of the other Italian and Dutch populations, which were smaller (mean value 470 vs. 421, 408 μm, respectively), the longer stylet of our population compared to that of the other Italian (16.7 vs. 15.0 μm) and Dutch populations (16.7 vs. 13.7 μm), and the slightly smaller a and c’ indices of the previously described population from Italy (29.9 and 14.3 vs. 25.9 and 12.6, respectively).

A study by [14] aimed to investigate the genetic relatedness among Mediterranean and European populations of *R. borealis* (=*R. macrosoma*) and revealed a distinction between populations from Greece and those from other European countries, despite only minor morphological differences. Our study shows a remarkable morphometric similarity between Italian (present study) and Greek populations, with a main difference concerning the stylet length (mean value 16.7 vs. 18.5 μm).

Morphometrics of six male specimens roughly reflect the differences observed for immature females, with shorter body lengths in previously described Italian and Dutch populations (mean value 466 vs. 437 and 418 μm, respectively) (Table S1). The latter also showed a shorter DGO (23.0 vs. 17.4, 17.2 μm) and smaller a ratio (30.3 vs. 27.6, 24.1). The spicule length was slightly longer in the other Italian population (22.9 vs. 20.6 μm), whereas smaller T values were found in our population vs. Spanish, Greek and Dutch populations (24.4 vs. 33.0, 31.8 and 39.7, respectively).

#### 2.1.2. Rotylenchulus Macrodoratus Dasgupta et al. 1968 [6]

**Figure 3:**
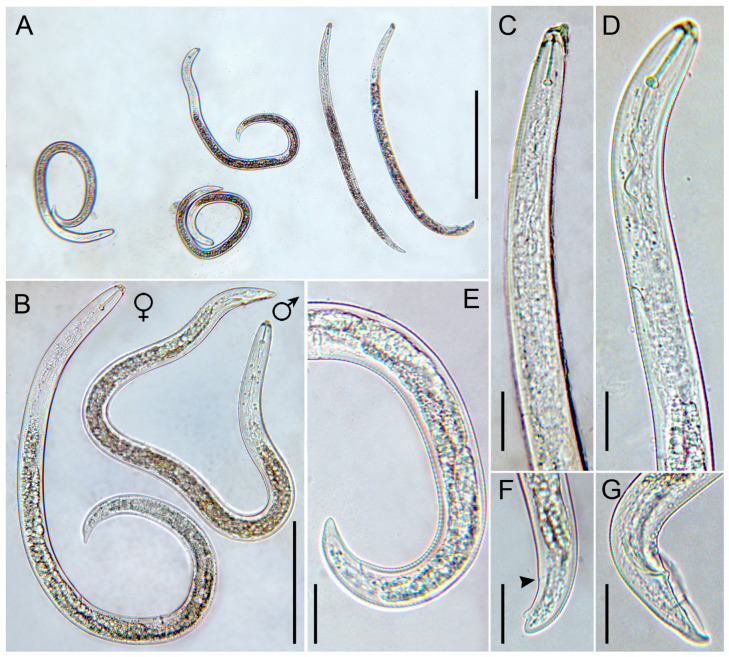
Light micrographs of *Rotylenchulus macrodoratus*. (**A**): Entire body of immature females, and a male (far right); (**B**): immature female and male entire body; (**C**): male pharyngeal region; (**D**): female pharyngeal region; (**E**): immature female vulval and tail region; (**F**): immature female aberrant tail (anus arrowed); (**G**): male tail. (Scale bars: (**A**) = 200 µm; (**B**) = 50 µm; (**C**–**G**) = 20 µm).

**Figure 4:**
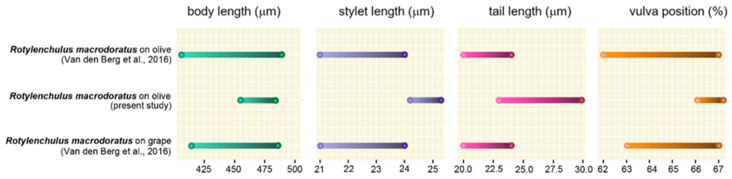
Schematic representation of the range of variations of four main diagnostic characters of immature females among *Rotylenchulus macrodoratus* population described in this work compared with previous populations described from Italy on olive and grape [13].

Seven immature females were measured, showing minor differences in stylet length, vulva position, and tail length compared to the previous descriptions of Italian populations (Figure 3, Table S2). The stylet was long and well-developed with anchor-shaped to rounded knobs, almost identical to that of the original description, but longer than that of the two Italian populations (25.1 vs. 22.2 and 22.1 μm, respectively) described in [13]. Figure 4 reports the schematic representation of the range of variations of the four main diagnostic characters of immature females of *R. macrodoratus* populations from Italy.

The vulva is located at 67%, slightly more anteriorly than in the original description (67 vs 71%) but more posterior in comparison with the other Italian populations (67 vs. 64.9 and 65.4%). The tail length is longer in our population with respect to other Italian and type populations (27.2 vs. 22–22–21 µm, respectively). The pharynx length is longer than other Italian populations (157 vs 130–136 µm). Further small differences concern the body length of Italian *R. macrodoratus* of present study, which is longer than that of other Italian populations (469 vs. 455–456–460 µm, respectively), and the b and c ratios, which are smaller than in all other Italian olive, grape and laurel populations (2.9 vs. 3.5–4.2, and 17.4 vs. 20.5–20.5–20, respectively). The c′ ratio corresponds with that of type population but is slightly higher than that of Italian olive and grape populations (2.4 vs. 2.1–2.2–2.4).

Five males in our population conform more closely to previous descriptions of other Italian populations (Table S2), with small differences concerning body length, shorter in our population, a slightly longer stylet (20.2 vs. 18.8, 19.3, 18.5 µm), longer tail (26.2 vs. 21, 22 µm), and smaller c ratio (18.9 vs. 23.6, 23.4, 21–28 respectively).

### 2.2. Principal Component Analysis

**Figure 5:**
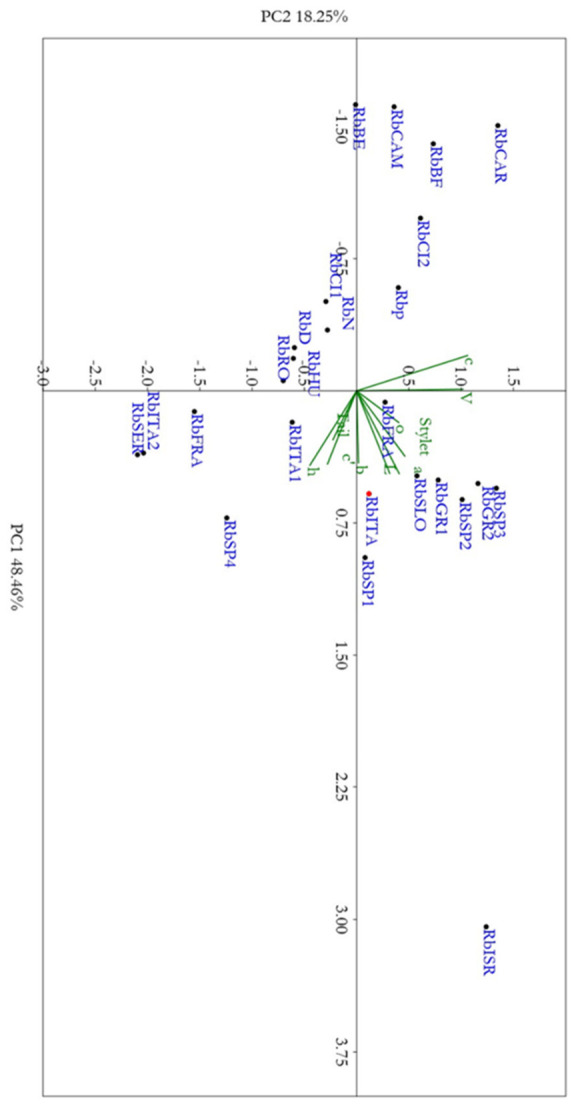
Principal component analysis (PCA) based on morphometric parameters to characterize *Rotylenchulus borealis* (Rb) from Italy and all over the world. Correlation biplot based on a PCA of the morphometric characters of *Rotylenchulus borealis* Italian population (red circle), compared with population previous described in the literature: RbITA (this study, Italy); RbISR [5], Israel) RbSP1 ([1], Spain); RbSP2, RbSP3 ([12], Spain); RbSP4 ([13], Spain); RbGR1, RbGR2 ([8], Greece); RbSER ([13], Serbia); RbIT1 ([5], Italy); RbIT2 ([13], Italy); RbFRA ([13], France); RbRO ([13], Romania); RbHU ([13], Hungary); RbD ([13], Germany); RbFRA1 ([5], France); RbSLO ([19], Slovakia); RbCI1, RbCI2 ([18], Cote d’Ivoire); RbBE ([18], Benin); RbCAR ([18], Central African Republic); RbCAM ([18], Cameroon); RbBF ([18], Burkina Faso).

The spatial distribution of *R. borealis* populations is shown in Figure 5. The PCA analysis was supported by Bartlett’s test of sphericity (χ^2^ = 210.42, df = 45, *p* < 0.001) and the KMO value (0.535), indicating non-random correlations among variables and an acceptable level of sampling adequacy. In the principal component analysis (PCA), an accumulated variability of 66.6% of the total variance was detected among *R. borealis* populations included in this study (Table S3). The PC1 axis is associated with specimens with lower values of body size (L), stylet length, o, and c that increased from the left side to the right side of the plot. The PC2 axis separates individuals according to tail length and h and c′ values; higher values of these parameters are in the upper part of the plot. The present Italian population RbITA grouped with the corresponding populations from temperate zones of Europe (other populations from Italy, Spain, Greece, France, Germany, Netherlands, Slovakia, Romania, and Hungary) showing a larger body size, tail, and stylet [9,14] compared to the populations from tropical areas of Africa (Côte d’Ivoire, Benin, Cameroon, Burkina Faso, and Central African Republic) with smaller more compact morphotypes, as reported by Germani [18]. Olive-associated populations (RbIta, Spain 1, 2,3, GR1 and 2, and ISR) of R. borealis along with the RbSLO population found on corn clustered together showing similar scores, with a consistent vulva position and hyaline tail length. Cereal (corn, wheat) and grape-associated populations from Central/Eastern Europe (UN) show distinct PC2 values, suggesting host-linked morphological variability. Tropical African populations on sweet potato tuber and rice show extreme PC2 values, consistent with high variability in morphometric characters as already described.

### 2.3. Molecular Characterization

**Figure 6:**
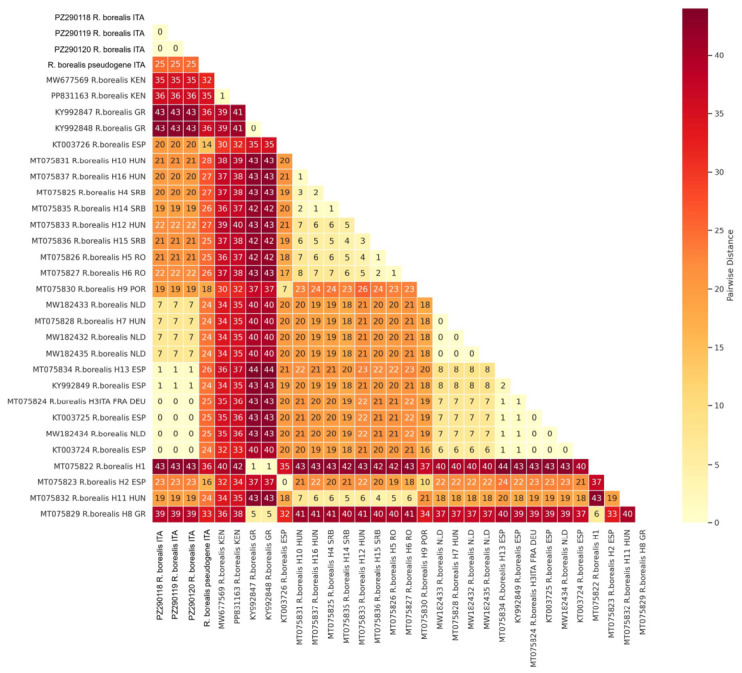
Pairwise distance heatmap of D2-D3 sequences of *Rotylenchulus borealis* populations included in this study. The number of base differences per sequence from between sequences are shown. The rate variation among sites was modeled with a gamma distribution (shape parameter = 1). All ambiguous positions were removed for each sequence pair. The upper triangle of the matrix is masked to enhance visualization.

The amplification of D2-D3 and ITS genes of *R. borealis* produced products of 762 and 982–987 bp, respectively. COI amplification produced two sequences of 418 and 428 bp, based on sequencing. The amplified D2-D3 domains of three individual specimens of *R. borealis* from organic cultivated olive in Italy were sequenced. Sequence analyses revealed that the new D2-D3 sequences showed 98–99% similarity with the corresponding sequences of *R. borealis* present in the database belonging to type A cluster, while they showed 95–96% similarity with the D2-D3 sequences of type B. Pairwise distances among D2-D3 sequences of *R. borealis* are shown in Figure 6. The number of nucleotide differences among all populations of *R. borealis* ranged from 2 to 112 nt. The Italian population of the current study differed by 1 to 44 bp from those belonging to type A and from those belonging to type B by 98 to 108 bp. The sequences MT775429 from India and OR957393 from Ethiopia differed by 44 to 93 bp from the Italian population. Two amplified products of *R. borealis* ITS from individual nematodes were cloned, and four clones were sequenced. Sequence analyses revealed the existence of two ITS paralogs in the *R. borealis* population studied, differing by 5 bp in length in the ITS1 and showing a 97% similarity (28 nt different and 7 gaps) to each other. These paralogs differed by nucleotide changes and varying numbers of short repetitive motifs within the sequence. Blast search revealed that the new ITS sequences of *R. borealis* showed 94-98% similarity (14–41 nt different; 3–14 gaps) with the corresponding sequences present in GenBank, all belonging to the type A group.

Two amplified fragments of COI from two individual nematodes were cloned, and four clones were sequenced. The sequence analysis of the COI clones revealed the presence of four haplotypes, three of them differed by 1/2 nt from each other, while the fourth one was shorter and differed from the previous by 35–36 nt (92% similarity, 10 gaps). It is noteworthy that the shortest haplotype was obtained from the same specimen of the longest one. The translation of COI sequences in amino acids revealed the use of the genetic code of FLATworm Mitochondrial Code (trans_table = 14) at NCBI; even the shortest haplotype seems to use FLATworm Mitochondrial Code (trans_table = 14), but still two stop codons, TAG, occurred and were coincident with tyrosine residue, as occurs in the sequences of other *Rotylenchulus* and nematode species [14].

**Figure 7:**
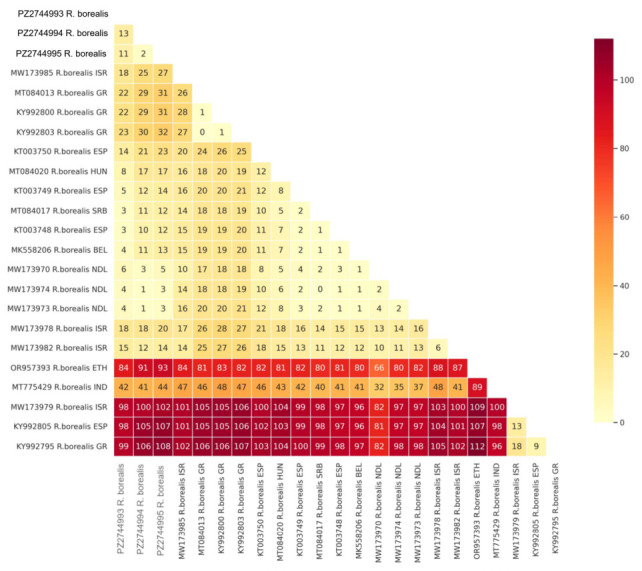
Heatmap of pairwise genetic distances among COI sequences of *Rotylenchulus borealis* included in this study. The number of base differences per sequence pair is shown within each cell. The rate variation among sites was modeled with a gamma distribution (shape parameter = 1). All ambiguous positions were removed for each sequence pair. The upper triangle of the matrix is masked to improve readability.

Pairwise distances among *R. borealis* COI sequences revealed that they differ by 0–44 bp (Figure 7). The longest Italian COI haplotypes differed by 0 to 7 bp with other COI sequences from Italy, France, Germany, Spain, Hungary, and the Netherlands, but they also differed by 19-43 nt with other haplotypes coi-H1, coi-H2, coi-H11 and coi-H8 from Spain, Hungary, and Greece, respectively (Figure 4). The COI sequences PP831163 and MW677569 from Kenya and KT992847-KT992848 from Greece differed by 36–43 bp, respectively. The shortest Italian COI haplotype differed by 14–40 bp among *R. borealis* COI sequences.

For *R. macrodoratus*, four individual specimens were amplified by using D2-D3 primers and directly sequenced. Intrapopulation differences between the obtained sequences were 84–98% (19–121 bp). Pairwise comparison of the D2D3 sequences of *R. macrodoratus* confirmed the high variability among populations ranging from 0 to 121 bp (Table S4). Interestingly, the D2-D3 haplotypes of the current population (H-IT23, H-IT18, H-IT59 and H-IT60) differed by 11–121 bp compared to other Italian populations of *R. macrodoratus*. Thus, four new Italian D2-D3 haplotypes were determined; three of them (H-IT18, H-IT59 and H-IT60) seem to be unique to the studied population.

### 2.4. Phylogeny

**Figure 8:**
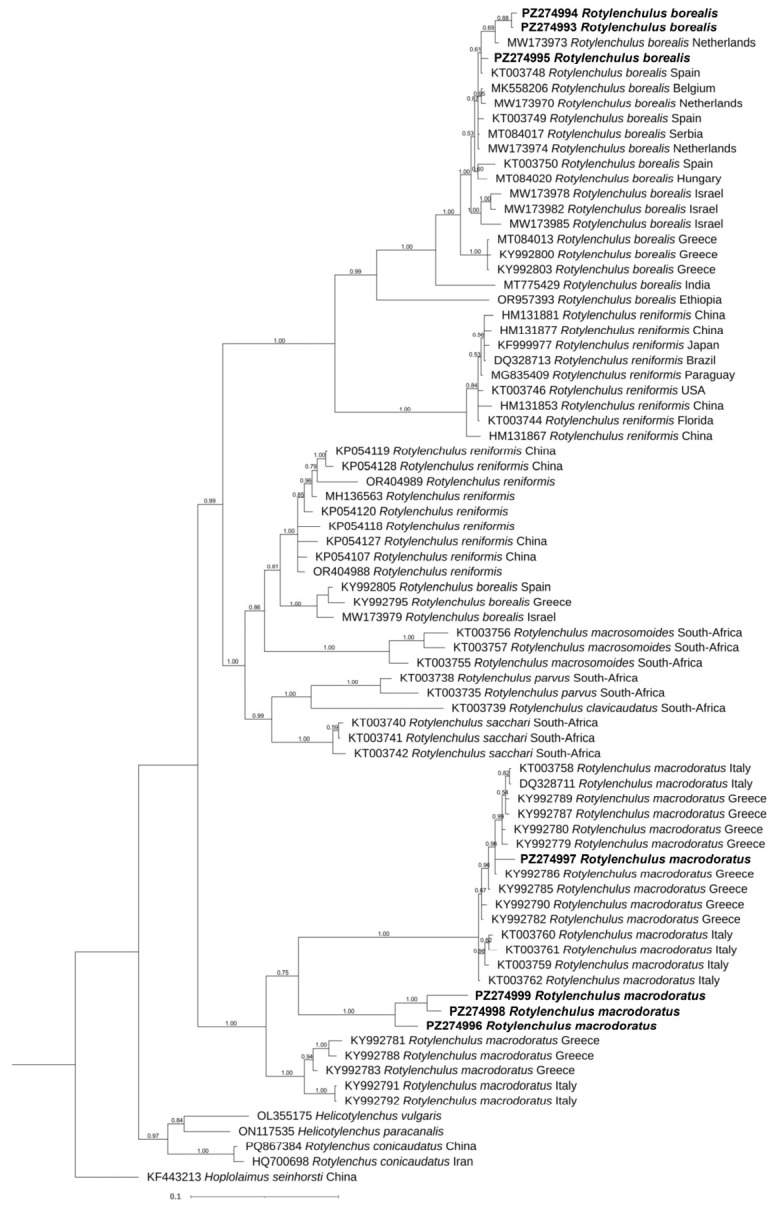
Phylogenetic relationships among *Rotylenchulus borealis* and *Rotylenchulus macrodoratus* populations using Bayesian 50% majority rule consensus tree as inferred from D2-D3 expansion domains of the 28S rRNA gene sequence alignment under a transversional with correction for invariable sites and a gamma-shaped distribution model (GTR+G). Posterior probabilities of more than 0.50 are given for appropriate clades. Newly obtained sequences in this study are shown in bold. The scale bar indicates expected changes per site.

The phylogenetic trees inferred by the BI method based on D2-D3, ITS and COI sequences, including the new ones obtained in the present study, are shown in Figure 6, Figure 7 and Figure 8, respectively. The D2-D3 tree, including the new sequences of *R. borealis* and *R. macrodoratus* from Italy, showed a topology like that reported in Palomares-Rius et al. [14]. Our *R. borealis* clustered with *R. borealis* type A from European countries and showed sister relationships with *R. reniformis* type A with high support (0.99). The new H-IT23 haplotype of *R. macrodoratus* clustered with *R. macrodoratus* type A, while the H-IT18, H-IT59 and H-IT60 subgrouped together revealing a sister relationship with *R. macrodoratus* type A.

The ITS tree revealed the occurrence of several subgroupings in the cluster of *R. borealis* type A and the new Italian sequences subgrouped with those from the Netherlands and Spain, while those from Greece, Israel, and Ethiopia subgrouped according to the geographical origin.

The COI phylogeny showed that all *R. borealis* haplotypes formed a supported clade in which five subgroupings occurred. Our COI sequences were in subgrouping I, but the subgroupings I, II and III included all COI haplotypes from western and eastern Europe with high support. Interestingly, subgrouping III included haplotypes from Spain, Portugal and the shortest one from our study. Subgrouping IV included only haplotypes from Kenya, and subgrouping V contained COI-H1 and COI-H8 haplotypes unique to Greece.

## 3. Discussion

The economic importance of *Rotylenchulus* genus is poorly investigated, as tree decline is often attributed to drought, nutrition, or other soil issues. The present study aimed to identify and characterize, morphologically and molecularly, two *Rotylenchulus* species found in the rhizosphere of traditional and organic olive orchards in central Italy and to evaluate the impact of the agronomic practices on reniform nematode composition. No species of *Rotylenchulus* were found in Sicily samples, suggesting that a broader survey is needed. Morphological and molecular analyses identified the two species as *R. borealis* and *R. macrodoratus* in olive groves in Central Italy. Recently, the occurrence of *R. reniformis*, *R. macrodoratus* and *R. borealis* in Italian olive groves have also been recorded [9,11,12,13] and poorly characterized only at morphological level. Thus, in our study, morphological measurements of the Italian *R. borealis* and *R. macrodoratus* populations are also provided. Figure 2 and Figure 4 show the morphometrics of both species agreeing quite well with previous descriptions. Few characters, as shown in Figure 2, are highly variable among *R. borealis* populations, as also observed by [14], while for *R. macrodorus* populations from Italy, the main characters overlapped, as shown in Figure 4. In addition, PCA confirmed the high variability among *R. borealis* populations, revealing that the present *R. borealis* population is placed close to those populations from European countries according to some characters (Figure 5). Sequencing of D2-D3 and ITS confirmed the existence of high intra-individual and intra-specific variation in both species. Additionally, the *R. macrodoratus* population from Italy revealed four D2-D3 haplotypes, H-IT18, H-IT23, H-IT59 and H-IT60. The sequence of the H-IT23 haplotype is identical to those of *R. macrodoratus* belonging to type A. But the H-IT18, H-IT59 and H-IT60 haplotypes showed an inter-individual variability ranging from 96 to 98% each other and an 84% variability (116 bp different) with H-IT23. The D2-D3 tree confirmed the high variability among *R. macrodoratus* populations. Additionally, the D2-D3 tree revealed that H-IT18, H-IT59 and H-IT60 haplotypes, forming a separated subgrouping closely related to the type A, are unique in the current Italian population (Figure 8). Furthermore, our results also prove that *R. macrodoratus* might have a monophyletic origin, as all D2-D3 sequences belonging to type A and B formed a well-supported clade (Figure 8).

**Figure 9:**
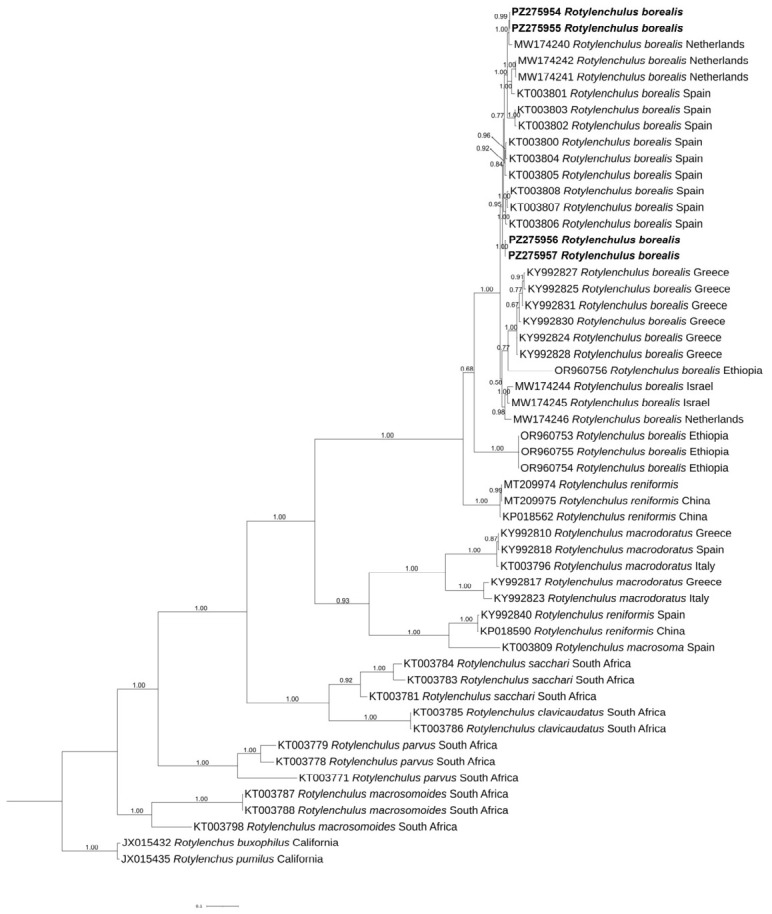
Phylogenetic relationships among *Rotylenchulus borealis* and *Rotylenchulus macrodoratus* populations using Bayesian 50% majority rule consensus tree as inferred from ITS sequence alignment under a transversional with correction for invariable sites and a gamma-shaped distribution model (GTR+G). Posterior probabilities of more than 0.50 are given for appropriate clades. Newly obtained sequences in this study are shown in bold. The scale bar indicates expected changes per site.

Both the D2-D3 and ITS phylogenetic trees of *R. borealis* confirmed the polyphyletic origin of this species, due to the existence of paralogues independent from geographical locations, stable in the genome and still evolving within the same individual (Figure 8 and Figure 9). Interesting, the occurrence of two different ITS paralogs in an individual nematode of the current population, with substantial sequence divergence, confirmed the intragenomic rRNA variability within individual nematodes of *R. borealis* [20,21]. This finding strongly suggested a different evolutionary history among cistrons or incomplete concerted evolution, previously described in *Rotylenchulus* species [22]. Often the paralogs can be interpreted for interspecific variation rather than intraspecific duplication. The presence of different paralogs In several *Rotylenchulus* spp. highlights that gene duplication could contribute to the adaptation to different environments and plant hosts as observed in *Meloidogyne* spp. [22].

**Figure 10:**
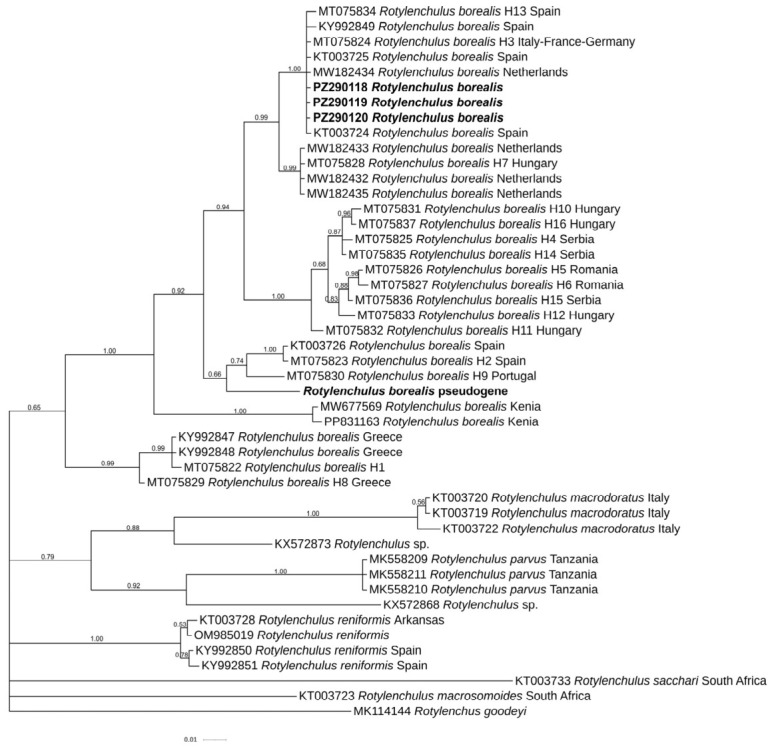
Phylogenetic relationships among *Rotylenchulus borealis* and *Rotylenchulus macrodoratus* populations using Bayesian 50% majority rule consensus tree as inferred from mitochondrial COI sequence alignment under a transversional with correction for invariable sites and a gamma-shaped distribution model (GTR+G). Posterior probabilities of more than 0.50 are given for appropriate clades. Newly obtained sequences in this study are shown in bold. The scale bar indicates expected changes per site.

In the current study, the sequencing of several COI clones revealed four haplotypes differing in length and nucleotide variability. The long *R. borealis* COI haplotypes used the alternative flatworm mitochondrial genetic code, while the short still contained two stop codons TAG, probably coding for tyrosine as does TAA stop codon in *Radopholus similis* [14,23,24]. As the same specimen contained both long and short haplotypes, this finding suggested the existence of heteroplasmic mitochondrial DNA or the existence of a mitochondrial COI pseudogene. The phylogenetic analysis based on COI revealed the clustering of all COI haplotypes of *R. borealis* and the occurrence of COI haplotype diversity among different geographical populations (Figure 10). These results clearly confirmed a higher mutation rate in mitochondrial DNA of *R. borealis,* as already reported in other nematodes [25,26].

## 4. Materials and Methods

### 4.1. Nematode Sampling, Extraction, Processing, and LM Pictures

Sampling was carried out in 2023 in nine olive orchards in Central and insular regions of Italy (Lazio, Tuscany, and Sicily) during the flowering period. Thirty-six soil samples and roots were collected in plastic bags to avoid water loss and stored at 4 °C until processing. Nematodes were extracted, using the centrifugal flotation method [27]. The two reniform nematodes, *R. borealis* and *R. macrodoratus*, were present in high densities (70 nematodes/100 cm^3^ soil and 85 nematodes/100 cm^3^ soil, respectively).

Specimens for microscopic observation were handpicked and killed by gentle heat, fixed in a solution of 4% of formaldehyde + 1% propionic acid, and processed in glycerol according to Seinhorst’s method in [28]. Glycerin-infiltrated specimens were examined by light microscopy for diagnosis. Measurements were taken on specimens mounted on permanent slides. Pictures were taken with a Leica DFC 425 camera mounted on a Leitz Diaplan (Wetzlar, Germany) compound microscope with incorporated software ‘LAS^®^ v. 3.6.0’. Morphological identification was based on main selected diagnostic characters (Tables S1 and S2). Two *Rotylenchulus* populations were identified in this study at morphological and molecular levels (Figure 1, Figure 2, Figure 3 and Figure 4).

### 4.2. Statistical Analysis

Principal component analyses (PCA) were performed to evaluate the distribution of all *R. borealis* populations according to ten morphological traits: body length (L), De Man indices (a, b, c, c′), vulva position (V), stylet length (Stylet), tail length (Tail), and hyaline tail terminus (H). A PCA was performed using PAST software v. 4.03 (Paleontological Statistics) [28] using standardized variables (z scores). High variability was observed for the components (PC1 and PC2) compared to the other components. The cumulative variance for PC1 and PC2 was 66.6%. The morphometric measurements of other species were taken from the original and previous descriptions in literature [2,6,9,13,14,18,19,29]. Before multivariate analysis, morphometric variables were log transformed to reduce skewness. PCA was performed using a correlation matrix, as variables were measured on different scales. The suitability of the dataset for PCA was assessed using Bartlett’s test of sphericity and Kaiser–Meyer–Olkin (KMO). The scores values were calculated for each species based on the principal components, and the scores for the first two components were used to form a two-dimensional plot (F1 and F2) of each isolate based on the eigenvalues given by the software (Figure 5, Table S3).

### 4.3. DNA Extraction and PCR

Ten individual nematodes for each species were crushed with a sterile micro-spatula under a stereomicroscope, as described by De Luca et al. [30]. Crude DNA from each individual nematode was directly used for PCR amplification. The D2A-D3B expansion segments of 28S rRNA gene was amplified using the primers D2A (5′-ACAAGTACCGTGGGGAAAGTTG-3′) and D3B (5′-TCGGAAGGAACCAGCTACTA-3′) [31]; the ITS1-5.8S-ITS2 regions were amplified using the forward primer TW81 (5′-GTTTCCGTAGGTGAACCTGC-3′) and the reverse primer AB28 (5′-ATATGCTTAAGTTCAGCGGGT -3′) [32]; the portion of the mitochondrial cytochrome oxidase c subunit 1 (*mt*COI) gene was amplified with this primer set: COI-F1 (5′-CCTACTATGATTGGTGGTTTTGGTAATTG-3′) and COI-R2 (5′-GTAGCAGCAGTAAAATAAGCACG-3′) [33]. PCR cycling conditions used for amplification were an initial denaturation at 94 °C for 5 min, followed by 35 cycles of denaturation at 94 °C for 50 s, annealing at 55 °C for 50 s and extension at 72 °C for 1 min and a final step at 72 °C for 7 min. The size of the amplification products was determined by comparison with 100 bp molecular weight ladder (Fermentas, St. Leon-Rot, Germany) following electrophoresis of 10 μL on a 1% agarose gel. PCR products of the D2-D3 expansion domains, the ITS containing region, and the mitochondrial COI, from individual nematodes, were purified using the protocol suggested by the manufacturer (High Pure PCR elution kit, Roche, Germany). Purified ITS and COI fragments were cloned with pGEM-T Vector System II kit (Promega, Madison, WI, USA). Positive clones were sent for sequencing in both directions with the primers given above or M13 forward and M13 reverse primers at MWG-Eurofins in Germany.

### 4.4. Estimations of Evolutionary Divergence Between Sequences

The number of nucleotide differences among the D2-D3 expansion domains of 28S rRNA gene and COI sequences was determined. The D2-D3 analyses of *R. borealis* and *R. macrodoratus* involved 23 nucleotide sequences each, while COI analysis of *R. borealis* involved 32 nucleotide sequences. All positions encompassing gaps and missing data were excluded. The pairwise distances were performed in MEGA 12 software package [34]. Heatmaps for pairwise distances were generated with R (version 4.5.2; R Core Team, Vienna, Austria) software using the packages ggplot2, reshape2, and RColorBrewer.

### 4.5. Phylogenetic Analysis

The newly obtained D2-D3, ITS and partial *mt* COI sequences were compared with those of other nematode sequences available in the GenBank database using the BLAST homology search program (https://blast.ncbi.nlm.nih.gov/Blast.cgi, accessed on 27 April 2026). The sequences were aligned using Clustal Omega (http://lilab2.sysu.edu.cn/Tools/msa/clustalo/, accessed on 27 April 2026) [35] and edited by using BioEdit [36]. Outgroup taxa for each dataset were chosen according to previously published data [14,15].

Phylogenetic trees were generated using the Bayesian inference method as implemented in the program Mr Bayes 3.1.2 [37]. The best fitting model of sequence evolution was determined for each dataset separately using the Akaike information criterion (AIC) implemented in jModeltest 2.1.10 [38,39]. The GTR+ G model was selected for all markers.

Bayesian analyses employing Markov chain Monte Carlo (MCMC) were carried out using MrBayes 3.1.2 [40]. Four MCMC chains were run simultaneously for 1 × 106 generations. Trees were sampled at intervals of 1000 generations. Two independent runs were performed, and after confirming the convergence of runs, the first 25% of generations were discarded as burn-in, with the remaining topologies used to generate a 50% majority-rule consensus tree and visualized using iTol version 6 [41].

## 5. Conclusions

In conclusion, our study confirms the occurrence of intragenomic rRNA variability in both *Rotylenchulus* species studied and a higher mutation rate of the COI gene. Moreover, our study confirmed the occurrence of intragenomic rRNA variability in individual specimens of *R. borealis,* suggesting different evolutionary rates inside the genome and between chromosomes. Further studies are needed to evaluate the potential effect of different rRNA variants as a form of functional flexibility and evolution in the genome of reniform nematodes.

In addition, our study revealed the occurrence of two distinct variants of COI in the same specimen of *R. borealis*, one of them still containing the stop codon that can represent a mitochondrial COI pseudogene.

## Data Availability

The data obtained during the current study are available from the corresponding author on request.

## Supplementary Materials

The following supporting information can be downloaded at https://www.mdpi.com/article/10.3390/plants15101423/s1, Table S1: Mean values, obtained from the literature, for each population used in PCA analysis; Table S2: Pairwise distance of D2D3 *R. macrodoratus* populations included in this study. The number of base differences per sequence from between sequences are shown. The rate variation among sites was modeled with a gamma distribution (shape parameter = 1). All ambiguous positions were removed for each sequence pair. Table S3. Mean values obtained from the literature, for each population used in PCA analysis. Table S4. Pairwise distance D2D3 *R. macrodoratus*.
