# Supplementary material for: Intragenomic rDNA Variants Identified in Rotylenchulus borealis and R. macrodoratus Populations Associated with Olive Groves in Italy
Source: Plants (Basel). 2026 May 7;15(10):1423. doi: 10.3390/plants15101423 (PMC13211144; doi:10.3390/plants15101423)
Supplement: Supplementary file 1 [file plants-15-01423-s001.zip › plants-4254805-supplementary.pdf]

Table S1. Morphometrics of immature females and males of *Rotylenchulus borealis* from central Italy (present study) compared with selected Mediterranean populations and type population. All measurements are in micrometers and in the form mean ± SD (range).

**Reference**	Present study<br>Italy		Castillo et al. (2003)<br>Spain		Palomares-Rius et al. (2018)<br>Greece		Palomares-Rius et al. (2021)<br>Italy		Palomares-Rius et al. (2021)<br>Netherlands, paratype	
host	Olive		Wild olive		Olive		Corn		Grasses	
**Character/Ratio**	Immature females	Males	Immature females	Males	Immature females	Males	Immature females	Males	Immature females	Males
n	11	6	12	11	10	5	10	10	3	5
L	470 ± 25.1<br>(431-501.5)	466.4 ± 16.5<br>(449.5-490.3)	453 ± 28.0<br>(408-510)	467 ± 13<br>(449-495)	467 ± 27.0<br>(432-506)	468 ± 28.2<br>(433-503)	427 ± 11<br>(411-441)	436.7 ± 28<br>(405-483)	408 ± 24<br>(381-424)	418 ± 12<br>(404-435)
a	29.9 ± 2.3<br>(27.2-34.2)	30.3 ± 2.3<br>(27.6-33.8)	29.8 ± 2.1<br>(26.3-34.2)	31.5 ± 2.1<br>(27.5-34.0)	28.9 ± 1.9<br>(26.1-31.6)	30.8 ± 2.1<br>(27.1-32.0)	25.9 ± 1.7<br>(23.0-28.0)	27.6 ± 1.8<br>(25.3-31.2)	27.9 ± 2.3<br>(25.4-29.9)	24.1 ± 2.8<br>(21.6-28.9)
b	3.2 ± 0.3<br>(2.8-3.6)	3.4 ± 0.1<br>(3.2-3.5)	3.9 ± 0.3<br>(3.5-4.4)	4.7 ± 0.7<br>(3.5-5.2)	3.6 ± 0.3<br>(3.3-4.3)	3.4 ± 0.3<br>(3.1-3.6)	3.6 ± 0.4<br>(2.9-4.2)	3.9 ± 0.2<br>(3.0-3.3)	4.1 ± 0.2<br>(3.9-4.2)	3.8 ± 0.2<br>(3.4-3.9)
c	14.3 ± 1.1<br>(12.6-16.0)	14.0 ± 0.9<br>(12.9-15.1)	13.5 ± 1.3<br>(11.7-16.8)	13.9 ± 0.6<br>(13.1-15.0)	15.3 ± 0.9<br>(13.8-17.0)	14.7 ± 0.9<br>(13.1-15.4)	12.6 ± 0.8<br>(11.8-14.5)	13.2 ± 0.9<br>(12.3-14.6)	14.9 ± 0.5<br>(14.6-15.5)	14.7 ± 1.0<br>(13.6-16.1)
c'	3.2 ± 0.3<br>(2.8-3.8)	3.2 ± 0.2<br>(3.0-3.4)	3.7 ± 0.5<br>(2.8-4.4)	3.2 ± 0.4<br>(2.6-3.9)	3.3 ± 0.3<br>(2.8-4.0)	3.0 ± 0.2<br>(2.8-3.3)	3.6 ± 0.2<br>(3.2-3.8)	3.1 ± 0.1<br>(3.0-3.3)	2.8 ± 0.2<br>(2.6-2.9)	2.8 ± 0.1<br>(2.6-2.9)
V or T	62.0 ± 2.4<br>(57.8-66.2)	24.4 ± 2.0<br>(21.1-26.0)	62 ± 2<br>(59-64)	33.0 ± 5.0<br>(25-42)	61.4 ± 2.0<br>(58.0-64.0)	31.8 ± 1.3<br>(30.0-33.0)	60.1 ± 1.0<br>(58.0-61.8)	23.4 ± 2.1<br>(21.5-26.0)	62.2 ± 1.0<br>(61.0-63.0)	39.7 ± 13.5<br>(23.4-56.4)
D.G.O.	24.9 ± 2.5<br>(19.6-27.5)	23.0 ± 0.8<br>(22.3-24.3)	25 ± 2.0<br>(22-27)	23 ± 2.0<br>(19-26)	23.8 ± 1.5<br>(21.0-26.0)	20.8 ± 1.3<br>(20.0-23.0)	17.7 ± 1.5<br>(16.0-20.0)	17.4 ± 1.0<br>(16.0-20.0)	17.0 ± 1.0<br>(16.0-18.0)	17.2 ± 0.8<br>(16.0-18.0)
Stylet lenght	16.7 ± 0.6<br>(15.9-17.5)	13.9 ± 0.8<br>(13.0-15.1)	16 ± 1.0<br>(15-18)	14 ± 1.0<br>(12-15)	18.5 ± 1.7<br>(16.0-21.0)	14.6 ± 0.5<br>(14.0-15.0)	15.0 ± 0.7<br>(14.0-16.0)	14.2 ± 0.4<br>(13.5-15.0)	13.7 ± 0.6<br>(13.0-14.0)	12.6 ± 0.5<br>(12.0-13.0)
Excretory pore to anterior end	87.4 ± 2.8<br>(84.3-90.7)	85.4 ± 3.3<br>(81.0-89.2)	91 ± 3.0<br>(85-98)	89 ± 4.0<br>(81-96)	91.6 ± 4.9<br>(84.0-99.0)	88.8 ± 4.5<br>(83.0-95.0)	-	-	-	
Pharynx length	145.9 ±11.0<br>(131.8-160.4)	138.5 ± 6.1<br>(129.6-146.2)	161 ± 15<br>(138-181)	125 ± 13<br>(110-157)	129.2 ± 9.3<br>(114.0-140.0)	138.4 ± 5.9<br>(132.0-146.0)	-	-	-	
Diam. at mid-body	15.8 ± 1.0<br>(14.5-17.9)	15.5 ± 1.3<br>(14.0-16.9)	15 ± 1.0<br>(14-17)	15 ± 1.0<br>(14-18)	16.2 ± 0.9<br>(15.0-18.0)	15.2 ± 0.8<br>(14.0-16.0)	-	-	-	
Diam. at anus	10.5 ± 0.8<br>(9.2-11.5)	10.5 ± 1.3<br>(9.1-12.3)	10 ± 1.0<br>(8-11)	11 ± 1.0<br>(9-12)	9.4 ± 1.2<br>(8.0-12.0)	10.6 ± 1.1<br>(9.0-12.0)	-	-	-	
Tail length	33.0 ± 2.2<br>(29.9-36.4)	33.4 ± 2.2<br>(31.2-36.8)	34 ± 4.0<br>(26-40)	34 ± 2.0<br>(30-36)	30.6 ± 2.8<br>(27.0-36.0)	31.4 ± 0.9<br>(30.0-32.0)	33.9 ± 2.0<br>(30.0-36.5)	33.6 ± 1.3<br>(32.0-36.0)	27.3 ± 1.5<br>(26.0-29.0)	28.6 ± 2.3<br>(26.0-32.0)
h	10.1 ± 1.6<br>(7.4-11.5)	9.7 ± 0.7<br>(9.0-10.6)	10 ± 1.0<br>(9-12)	11 ± 2.0<br>(8-14)	10.4 ± 1.1<br>(9.0-12.0)	10.2 ± 1.3<br>(9.0-11.0)	12.5 ± 1.7<br>(10.5-15.0)	8.5 ±0.9<br>(7.0-10.0)	10.0 ± 1.0<br>(9.0-11.0)	9.9 ± 0.7<br>(9.0-11.0)
Spicule length	-	20.6 ± 1.4<br>(19.2-22.6)	-	22 ± 2.0<br>(19-25)	-	21.8 ± 1.3<br>(20.0-23.0)	-	22.9 ± 0.7<br>(22.0-24.0)	-	21.2 ±0.8<br>(20.0-22.0)
Gubernaculum lenght	-	8.4 ± 1.0<br>(7.5-10-0)	-	9 ± 1.0<br>(8-10)	-	9.2 ± 0.8<br>(8.0-10.0)	-	7.7 ± 0.3<br>(7.0-8.0)	-	7.0 ± 0.7<br>(6.0-8.0)

Table 2. Morphometrics of immature females and males of *Rotylenchulus macrodoratus* from central Italy (present study) compared with previously described Italian and type populations. All measurements are in micrometers and in the form mean ± SD (range).

**Immature females**	**Present study Italy**		**Italy [13]**		**Italy [13]**		**Italy [6]**	
	Olive		Olive		Grape		Laurel	
	Immature females	Males	Immature females	Males	Immature females	Males	Immature female	Male
n	7	5	9	5	7	3	Holotype	12
L	469 ± 9.0 (455-479)	492.3 ± 19.8 (468-515)	455 ± 30.0 (407-489)	503 ± 36.7 (452-540)	456 ± 24.9 (414-487)	507 ± 26.2 (478-529)	460	450-530
a	24.8 ± 1.1 (23.7-26.7)	28.0 ± 1.1 (26.3-29.4)	26.1 ± 1.5 (22.6-27.4)	31.4 ± 1.2 (30.1-33.1)	26.4 ± 0.7 (25.5-27.4)	31.7 ± 1.4 (30.2-33.1)	28	27 - 32
b	2.9 ± 0.6 (2.9-3.0)	3.2 ± 0.1 (3.1-3.3)	3.5 ± 0.4(2.9-4.0)	3.8 ± 0.5 (3.1-4.6)	3.5 ± 0.3 (2.9-4.0)	3.7 ± 0.1 (3.6-3.8)	4.2	3.8-4.6
c	17.4 ± 2.1 (15.7-20.6)	18.9 ± 2.1 (16.3-21-5)	20.5 ± 1.3 (18.5-22.2)	23.6 ± 2.6 (20.5-27.0)	20.5 ± 1.1 (18.8-21.6)	23.4 ± 1.7 (21.7-25.2)	20	21-28
c'	2.4 ± 0.4 (1.9-2.7)	2.2 ± 0.3 (1.8-2.8)	2.2 ± 0.1(2.0-2.4)	2.1 ± 0.2 (1.8-2.2)	2.2 ± 0.1 (2.0-2.4)	2.1 ± 0.1 (2.0-2.2)	2.4	-
o	58.4 ± 2.9 (55.6-62.6)	85 ± 4.7 (80.4-90.4)	68.0 ± 6.8 (61.9-81.8)	91.6 ± 2.5 (89.5-94.4)	69.7 ± 6.8 (62.5-81.8)	91.5 ± 2.6 (90.0-94.4)	64	-
D.G.O.	14.6 ± 0.5 (14.3-15.4)	16.34 ± 0.6 (15.6-17.0)	15.1 ± 1.6 (13.0-18.0)	17.2 ± 0.8 (16.0-18.0)	15.4 ± 1.6 (14.0-18.0)	17.7 ± 0.6 (17.0-18.0)	-	-
V or T	67.0 ± 0.6 (66.3-67.7)	24.6 ± 1.1 (23.4-26)	64.9 ± 1.6 (62.0-67.0)	25.0 ± 2.7 (21.7-28.0)	65.4 ± 1.3 (63.0-67.0)	25.2 ± 2.1 (23.8-27.6)	71	-
Stylet length	25.1 ± 0.6 (24.5-25.7)	20.2 ± 0.6 (19.6-21)	22.2 ± 1.2 (21.0-24.0)	18.8 ± 1.3 (17.0-20.0)	22.1 ± 1.1 (21.0-24.0)	19.3 ± 1.2 (18.0-20.0)	25	17-20
Excretory pore to anterior end	94.1 ± 4.5 (91-102)	95.7 ± 4.2 (91-101)	91 ± 6.6 (81-102)	92.0 ± 7.1 (80-98)	90.0 ± 6.6 (81-100)	94.0 ± 2.5 (91-96)	100	-
Pharynx length	157.3 ± 2.9 (154-162)	152.3 ± 3.4 (149.2-157)	131 ± 9.3 (116-142)	134 ± 10.9 (118-144)	130 ± 10.0 (116-142)	136 ± 7.2 (128-141)	-	-
Diam. at mid-body	18.9 ± 0.9 (17.5-20)	17.5 ±1.4 (15.9-19.2)	17.4 ± 0.9 (16.0-18.0)	16.0 ± 1.0 (15.0-17.0)	17.3 ± 1.0 (16.0-18.0)	16.0 ± 1.0 (15.0-17.0)	-	-
Diam. at anus	11.3 ± 0.6 (10.7-12.2)	11.5 ± 0.8 (10.2-12.4)	10.1 ± 0.8 (9.0-11.0)	10.4 ± 0.5 (10.0-11.0)	10.0 ± 0.8 (9.0-11.0)	10.3 ± 0.6 (10.0-11.0)	-	-
Tail length	27.2 ± 3.3 (23-29.8)	26.2 ± 2.6 (23.4-29.4)	22.0 ± 1.1 (20-24)	21.0 ± 0.9 (20-22)	22.0 ± 1.3 (20-24)	22.0 ± 0.6 (21-22)	21	-
Spicule length	-	20.1 ± 0.3 (19.7-20.5)	-	20.0 ± 1.0 (19.0-21.0)	-	20.7 ± 0.6 (20.0-21.0)	-	20
Gubernaculum length	-	9.3 ± 0.7(8-10)	-	9.4 ± 0.9 (8.0-10.0)	-	9.7 ± 0.6 (9.0-10.0)	-	7

Table S3. Mean values obtained from literature, for each population used in PCA analysis.

	**L**	**a**	**b**	**c**	**c'**	**V**	**o**	**Stylet**	Tail
Rbp	408	27.9	4.1	14.9	2.8	62.2	124.4	13.7	27.3
RbITA	470	28.8	3.3	14.2	3.2	61.1	149	16.6	33.4
RbISR	640	37	4.75	14	4.35	64	143	18	39
RbSP1	453	29.8	3.9	13.5	3.7	62	152	16	34
RbSP2	476	30.1	3.7	15.6	3.3	61.5	135.8	17.8	30.8
RbSP3	484	29.7	3.7	15.4	3.1	62.6	138	17.3	31.5
RbSP4	461	27.6	3.1	12.6	3.5	61.1	127.4	16.3	37.1
RbGR1	467	28.9	3.6	15.3	3.3	61.4	129.8	18.5	30.6
RbGR2	488	29.7	3.7	15.4	3.1	62.4	128.8	17.4	31.8
RbSER	425	26.2	3.5	12.6	3.7	59.9	120	14.9	33.8
RbITA2	427	25.9	3.6	12.6	3.6	60.1	118	15	33.9
RbFRA	428	26.3	3.6	12.8	3.2	60.5	120.1	14.9	33.5
RbRO	435	27.3	3.7	14	3	60.3	124	15.3	31
RbHU	430	27	3.7	14.1	3	60.6	121.3	15.1	30.6
RbD	422	27.3	3.6	14	3	60.6	125	14.9	30.1
RbN	427	27.5	3.2	13.8	3	61.9	119.3	14.8	30.7
RbFRA	415	28	3.9	14	3.1	62	166	14	29.6
RbITA1	415	27	3.95	13.5	3.25	61.5	146	14	30.7
RbSLO	428	29	4.2	13.9	3.4	63	145	15.5	31
RbCI1	420	27	2.9	14.7	3.4	61	131	14	30
RbCI2	420	25	2.9	16.7	3.2	62	119	14	-
RbBE	330	23	2.7	14.2	2.7	63.6	134	13	-
RbCAR	390	25.2	2.8	15.5	2.7	63.7	112	15.5	-
RbCAM	370	22.6	2.6	15.5	3.2	62	139	13.6	-
RbBF	400	24.5	2.7	15.8	2.6	62	131	14.4	-

Table S4. Pairwise distance D2D3 *R. macrodoratus*.

	[1]	[2]	[3]	[4]	[5]	[6]	[7]	[8]	[9]	[10]	[11]	[12]	[13]	[14]	[15]	[16]	[17]	[18]	[19]	[20]	[21]	[22]	[23]
1] 23 *R. macrodoratus*																							
[2] R. 60	121																						
[3] R. 59	116	19																					
[4] 18D3b *Rotylenchulus*	116	36	28																				
[5] KT003758 *R. macrodoratus* ITA	11	106	101	110																			
[6] DQ328711 *R. macrodoratus* ITA	12	107	102	11	0																		
[7] KY992789 *R. macrodoratus* GR	14	118	112	111	2	2																	
[8] KY992787 *R. macrodoratus* GR	12	116	110	109	2	2	2																
[9] KY992780 *R. macrodoratus* GR	13	117	111	112	3	3	3	3															
[10] KY992779 *R. macrodoratus* GR	14	116	110	111	3	4	4	4	3														
[11] KT003761 *R. macrodoratus* ITA	20	120	115	113	12	10	12	12	15	16													
[12] KT003760 *R. macrodoratus* ITA	15	119	114	112	7	6	7	7	10	11	4												
[13] KT003759 *R. macrodoratus* ITA	14	116	111	109	6	5	8	8	9	10	6	3											
[14] KY992786 *R. macrodoratus* GR	9	115	110	110	4	5	5	5	4	5	13	8	7										
[15] KY992785 *R. macrodoratus* GR	12	118	113	111	7	8	6	6	7	8	10	7	8	3									
[16] KT003762 *R. macrodoratus* ITA	14	116	111	109	6	6	6	6	9	10	5	5	4	7	4								
[17] KY992790 *R. macrodoratus* GR	15	117	112	110	7	7	5	7	8	9	7	6	5	6	5	3							
[18] KY992782 *R. macrodoratus* GR	15	117	112	110	7	7	5	7	8	9	7	6	5	6	3	1	2						
[19] KY992791 *R. macrodoratus* ITA	105	89	82	77	98	100	100	98	101	100	100	100	99	99	97	96	98	97					
[20] KY992792 *R. macrodoratus* ITA	105	89	82	77	98	100	100	98	101	100	100	100	99	99	97	96	98	97	0				
[21] KY992783 *R. macrodoratus* GR	99	86	79	74	93	94	94	92	95	92	94	94	93	93	91	90	92	91	18	18			
[22] KY992781 *R. macrodoratus* GR	99	90	83	78	92	92	92	90	93	94	94	94	93	93	91	90	92	91	27	27	14		
[23] KY992788 *R. macrodoratus* GR	100	93	86	81	95	95	95	93	96	95	95	95	94	94	92	91	93	92	26	26	11	9	
